# COVID-19 prophylaxis in immunosuppressed patients: Beyond vaccination

**DOI:** 10.1371/journal.pmed.1003917

**Published:** 2022-01-28

**Authors:** Ivan Gentile, Nicola Schiano Moriello

**Affiliations:** Section of Infectious Diseases, Department of Clinical Medicine and Surgery, University of Naples Federico II, Naples, Italy

## Abstract

Ivan Gentile and Nicola Schiano Moriello discuss the potential of monoclonal antibody prophylaxis against COVID-19 infection in immunocompromised patients.

## Introduction

Since early 2020, the Coronavirus Disease 2019 (COVID-19) pandemic has caused hundreds of millions of cases and several million deaths worldwide [1]. The development of effective vaccination has substantially changed the course of the pandemic, and the introduction of mass vaccination policies in most high- and middle-income countries has drastically reduced the number of new cases, hospitalizations, and deaths. However, vaccination is not the only tool able to provide immune prophylaxis against COVID-19. Herein, we discuss the use of monoclonal antibodies in addition to vaccination in order to better protect vulnerable people, particularly those with immunosuppression.

### Reduced vaccine efficacy in immunocompromised patients

In most high-income countries, 4 vaccines have been authorized for primary prophylaxis against COVID-19. These vaccines afforded up to 95% protection against the disease in clinical trials. However, their efficacy tends to decline over time [2]. Moreover, COVID-19 vaccination was found to have suboptimal efficacy in immunocompromised patients, thus leaving a nonnegligible portion of these patients at risk of infection [3]. In particular, patients undergoing treatment with rituximab are at high risk of not developing a serological response to COVID vaccination [4]. Studies in Israel and the USA found that 40% and 44% of hospitalized vaccine-breakthrough cases, respectively, were immunocompromised patients [5,6]. Notably, the proportion of immunosuppressed adults in the USA is estimated to be about 4% of the population, and this number is likely to increase due to greater life expectancy, improved medical management, and the introduction of new immunosuppressive treatments [7].

Immunosuppression should be seen as a continuous spectrum of different conditions, with fully immunocompetent patients at one extreme and patients with completely impaired immune function at the other, interspersed with several degrees of immunosuppression that can be associated with one or more exacerbating factors (for instance, advanced age, chronic diseases, and congenital immunodeficiencies). Moreover, the use of targeted immunosuppressive therapies has induced different conditions, in which one or more specific branches of the immune system are nonfunctional while the others are almost untouched.

Some studies have shown that the strategy of administering a third dose of COVID-19 vaccine could benefit some groups of immunocompromised patients, such as recipients of solid-organ transplants or patients in hemodialysis [8]. However, this strategy is not effective in all patients: For instance, patients with B cell lymphoproliferative disease in treatment with rituximab or ibrutinib seem to respond only partially to COVID-19 vaccine, even after a third dose [9]. Therefore, several million people, although fully vaccinated with 3 doses, remain vulnerable to Severe Acute Respiratory Syndrome Coronavirus 2 (SARS-CoV-2) infection.

### Prophylaxis in immunosuppressed patients

Immune prophylaxis could be the answer. Patients unable to produce antibodies after antigen administration, or with a contraindication to vaccination, could receive preformed antibodies. Data on immunotherapy in SARS-CoV-2 infection could provide insights into the efficacy of this approach. Growing evidence supports the use of monoclonal antibodies to treat infected patients at a high risk of progression [10]. In randomized clinical trials, these agents had an efficacy of between 70% and 86% in reducing hospitalizations and death in high-risk patients. A real-world study carried out in the USA confirms these data: The risk of hospitalization was 82% lower in patients treated with monoclonal antibodies than in untreated patients [11].

However, monoclonal antibodies against SARS-CoV-2 remain underused [12]. The cost of monoclonal antibodies is probably one of the main barriers to their use. However, the upfront cost of monoclonal antibodies was found to be offset by the reduction of hospital admissions when used, with an overall reduction of costs [12]. Another barrier to the adoption of monoclonal antibodies could be the narrow timeframe in which they retain their maximum effect, thereby requiring administration as soon as possible after symptom onset. The time factor requires that patients should be diagnosed and referred to care in a very short time, thereby placing a heavy burden on an already overloaded healthcare system. To avoid these bottlenecks, we suggest administering monoclonal antibodies as post- or even as preexposure prophylaxis for vulnerable people. In a randomized controlled trial, the subcutaneous infusion of casirivimab/imdevimab within 96 hours of household contacts of a confirmed SARS-CoV-2 case led to a significant reduction in the chance of developing symptomatic disease versus placebo (relative risk reduction of 92.6% for symptomatic disease) [13]. In another study in which bamlanivimab was administered as postexposure prophylaxis to the residents and staff of 74 skilled nursing and assisted living facilities in the USA with at least one confirmed SARS-CoV-2 index case, there was a lower risk of developing mild or worse disease (odds ratio 0.43 [95% CI, 0.28 to 0.68]) versus placebo [14]. Regarding the use of monoclonal antibody against COVID-19 as preexposure prophylaxis, a randomized Phase III trial is ongoing to test the safety and efficacy of tixagevimab/cilgavimab to prevent COVID-19 in unvaccinated adults ≥18 years without prior SARS-CoV-2 infection. The trial enrolled 5,150 patients that were randomized in a 2:1 ratio to receive the active combination or placebo. Preliminary results show that a single 300-mg dose of 2 intramuscular injections reduced the risk of developing symptomatic COVID-19 by 77% (95% CI 46 to 90) compared to placebo after 6 months [15]. Based on these results, the US FDA has issued emergency use authorization for tixagevimab/cilgavimab for postexposure prophylaxis.

Notably, duration of the protection provided by monoclonal antibodies for preexposure prophylaxis depends also on their half-life. The half-life of antibodies currently available for SARS-CoV-2 infection is relatively long, and different antibodies have different half-lives depending on the type of Fc (effector) region (Table 1). The combination tixagevimab/cilgavimab has a significantly longer half-life versus other antibodies because its Fc region has been specifically engendered for this purpose. The antibodies’ minimum effective concentration is still to be determined as is the optimal interval between doses. Due to the potential neutralizing effect of monoclonal antibodies, even at low concentrations, the interval between one administration and another could exceed that of the antibody’s half-life. Moreover, a comparative analysis on the efficacy of the various monoclonal antibodies has yet to be performed, and the efficacy data from registration trials are not comparable because they were conducted in different populations.

**Table 1:** Half-lives of the main monoclonal antibodies with neutralizing activity against SARS-CoV-2.

Monoclonal antibody	Half-life
Casirivimab/Imdevimab (s.c.)	31.8/26.9 days [16]
Bamlanivimab/Etesevimab	17.6/25.1 days [16]
Sotrovimab (i.v.)	49 days [15]
Sotrovimab (i.m.)	Trial ongoing [16]
Tixagevimab/cilgavimab (i.v)	Approximately 90 days [17]

i.m., intramuscular; i.v., intravenous; s.c., subcutaneous.

## Discussion

The use of monoclonal antibodies for COVID-19 prophylaxis may be a promising strategy to limit infections, particularly in patients with contraindications to vaccine or with low rate of response to vaccination. As most of these people have an impaired immune function, prophylaxis with monoclonal antibodies may contribute to reduce the infection (which is often long-lasting) in such populations, and it may also limit viral circulation, and therefore prevent the selection of variants. Further studies are needed to verify the efficacy of this strategy. Moreover, it must be noted that in low-income countries, the upfront cost of monoclonal antibodies could simply be out of reach. In this context, to cover the expense of the treatment and to provide fast access to care, specific programs should be implemented, and, ideally, the treatment should be offered at cost price.

In conclusion, although several studies have proven the efficacy of monoclonal antibodies for the treatment and prophylaxis of SARS-CoV-2 infection, real-world data on the efficacy and safety of monoclonal antibodies for pre- and postexposure prophylaxis are still lacking. If confirmed by large real-world studies, the strategy described herein would add to the arsenal of weapons in the fight against COVID-19, by complementing vaccination in patients with impaired immune function.
